# Maintaining competency: a qualitative study of clinical supervision and mentorship as a framework for specialist paramedics

**DOI:** 10.29045/14784726.2018.12.3.3.10

**Published:** 2018-12-01

**Authors:** Andrew Hodge, Samuel Swift, John P. Wilson

**Affiliations:** Yorkshire Ambulance Service: Orcid ID: 0000-0002-2632-2249; University of Sheffield; University of Sheffield

**Keywords:** clinical competence, mentor, paramedic

## Abstract

**Introduction:**

The aim of this study was to explore the factors influencing the maintenance of clinical competence and the effectiveness of the specialist paramedic in the context of mentorship, from the specialist paramedic’s own perspective.

**Methods:**

Semi-structured interviews were conducted with eight specialist paramedics in four regions of one ambulance service. Thematic analysis and coding were used to explore the data and identify emergent themes.

**Results:**

The study identified three key themes: appropriate clinical exposure; support and development; and opportunity for reflection. A tailored clinical leadership and mentorship model is required to maintain competency and effectiveness of specialist paramedics. Participants valued a model that delivered support, development and role clarity. Experienced advanced practitioners as mentors and organisational commitment were highlighted as essential components.

**Conclusions:**

Mentorship is an essential training requirement in extended roles to maximise efficacy of complex care out of hospital, to maintain clinical competence and as a source of motivation and psychological support.

## Introduction

**Figure 1.**
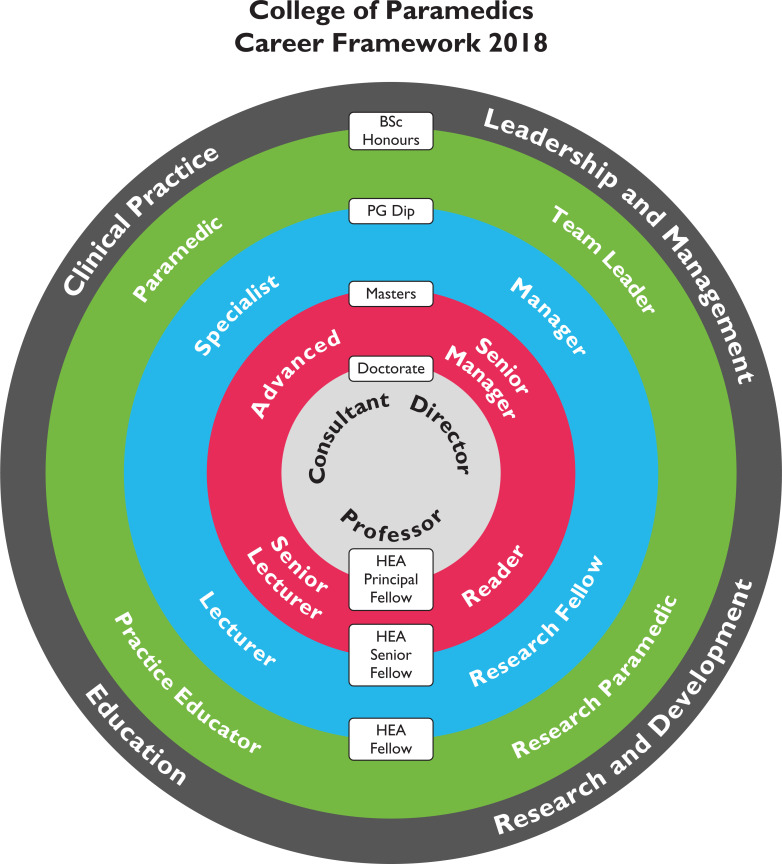
College of Paramedics career framework.

With the continuous increase in the demands placed upon NHS urgent and emergency care services, new ways of working have begun to emerge in order to cope with high patient volumes (NHS Institute of Innovation and Improvement, 2014). One of the ways that some ambulance services have responded to this is by implementing the College of Paramedics’ (2015) career framework (Figure 1). This framework aims to drive the development of the profession, providing paramedics with specialist and advanced knowledge and skills to provide more appropriate care to their patients.

Since 2014, the Yorkshire Ambulance Service (YAS) has commissioned the education of specialist paramedics, with a curriculum focused on lower acuity patients with minor illness, minor injury and long-term condition presentations. Part of the aim of this strategy is to avoid unnecessary emergency department (ED) attendances, since non-conveyance rates to EDs, while contentious, are one measure used to determine the effectiveness of ambulance services (O’Cathain et al., 2014).

The current model of operational clinical supervision is provided by clinical supervisors, who are paramedics with clinical leadership and first line managerial responsibilities. As a result, while all paramedics receive supervision for standard paramedic practice, those clinicians who have developed into specialist or advanced roles do not have clinical supervision tailored to their speciality.

While there is evidence relating to the positive impact of various types of paramedic practitioner roles in the community (Mason et al., 2007, 2012; O’Hara, O’Keefe, Mason, Coster, & Hutchinson, 2001; O’Keefe, Mason, & Knowles, 2014), little is known about how practitioners can remain competent to practise when they operate alone and independently in the out-of-hospital environment. One possible solution is the implementation of a clinical supervision framework delivered by advanced practitioners, including advanced paramedics, advanced clinical practitioners, doctors or advanced nurse practitioners.

Mentorship by peers may assist in the prevention of knowledge and skill decay. Yet with many mentorship models already developed, and the consensus that use of mentorship models is beneficial to clinicians (Gopee, 2015), it is not clear which model would provide the best fit for the specialist paramedic role.

The aim of this study was to explore the perceived factors that influence the effectiveness of the specialist paramedic role, with a focus on clinical leadership and mentorship. The primary objective was to identify a mentorship model to meet the needs of this relatively new specialism within the profession.

## Methods

From a pool of 130 specialist paramedics employed by YAS, eight were randomly selected (two from each of the four regions in Yorkshire) and invited to participate in the study. While a contingency was in place to allow for re-sampling, all eight of the initially approached paramedics elected to take part.

The interviews were designed and conducted by a university Master’s degree student with no prior knowledge of the paramedic profession, and supported by a senior paramedic clinician and a university supervisor. A subjective research design was developed from a phenomenological perspective, using semi-structured face-to-face interviews that were conducted in the participant’s workplace. The components and questions of the interviews were derived from a literature review to achieve the objective of understanding what a mentorship model should look like (Table 1).

**Table 1.** Interview topic guide.

Component	Questions
Background	Career history Qualified as a paramedic, and then as a specialist paramedic? Work experience in YAS Qualifications achieved and current Career ambitions
Current working practices	How are you able to maintain your clinical competence? Have you had previous experience of a mentor for your development? Was this positive or negative? What were the most successful characteristics of the mentor?
Proposed mentorship	What is the potential of having a mentor to maintain your clinical competency? What do you think a mentorship scheme for your role should look like? Frequency, where, when, who with? Are there any factors that could hinder effectiveness? Do you think that mentors should be appropriately trained? Would you prefer a formal or informal mentorship model? To what extent might mentorship help staff to feel valued? What would be the largest benefits for your role in being mentored? Consider professional and personal elements.

Interviews were audio recorded and transcribed for thematic analysis. Themes were generated using coding from the data, with repeated analysis allowing for the refinement of codes as new themes emerged. These themes provided the findings and concepts for this study, which was reported using the COREQ checklist for qualitative studies (Tong et al., 2007).

## Results

### Demographic data

All participants were aged over 40, with four participants in the age range 40–45 years and the remaining four in the age range 46–50 years. Most participants were male, with only two female participants. The numbers of years’ experience as a paramedic ranged from 11 to 39 years giving a total of 155 years’ combined experience. Table 2 shows the years of paramedic experience of each participant.

**Table 2.** Participants’ years of paramedic experience.

	SP1	SP2	SP3	SP4	SP5	SP6	SP7	SP8
Years’ experience	13	14	20	11	22	39	19	17

### Thematic analysis

Three main themes were identified from the thematic analysis of the data relating to the enabling of participants to be effective specialist paramedics:

- appropriate clinical exposure;
- support and development; and
- opportunity for reflection.

Following these themes, a section on mentorship as a concept related to their role was discussed and used to identify an appropriate mentorship model that was perceived to meet the needs of the specialist paramedic.

#### Appropriate clinical exposure

Clinical exposure to the most appropriate 999 calls relevant to the specialist paramedic role was the principle most often discussed. This was considered relevant to the maintenance of a specialist paramedic’s competence, and seen to allow the clinician to practise their skills more frequently when allocated an appropriate caseload.

We don’t use our skills very often, we are deskilling and losing confidence generally.

The range of clinical presentations facing the specialist paramedic attending 999 calls was considered so broad that participants felt their skills needed to encompass the management of acutely unwell patients, in addition to those with urgent care presentations. A mentorship model was thought to be one solution in addressing these concerns.

My scope of practice is just getting bigger and bigger. Someone calls 999 and it would still be me there first, and I’ve got to have a brief knowledge of what’s going on. You’ve got to have mentorship to keep you on top of your game with all this.

#### Support and development

The current clinical supervision model within YAS was identified as inadequate for the specialist paramedics’ new scope of practice, given the current model’s focus on the traditional paramedic role. This meant that there was no consideration of the specialist paramedics’ urgent care competencies. Advanced practitioners were thought to be the only professional group able to provide mentorship in order to teach and transfer knowledge.

Currently we do have some period of assessment through clinical supervisors but that is infrequent, and is obviously not tailored to the specialist paramedic role.

#### Opportunity for reflection

A general consensus emerged regarding the challenges in seeking protected time to learn and reflect. Participants felt they had no alternative but to seek additional employment, as a means to gain clinical development and maintain their specialist paramedic urgent care skills.

In addition, the inability to reflect upon stressful clinical cases due to time pressures driven by high 999 call demand was highlighted by most participants as a source of psychological stress, and impacting upon the clinical effectiveness of their role.

Before you’ve even got the chance to think, another call comes through and you’re off, and while you forget the job you’ve just done it does stay up here [pointing to head] and I think it’s showing now.

#### Mentorship from the specialist paramedic’s perspective

Most participants expressed a desire for individual face-to-face mentorship rather than group events. Spending time with an advanced practitioner in clinical practice ‘on the road’ was viewed as the most beneficial form of mentorship and supervision, allowing the specialist paramedic to observe and be observed, and providing an opportunity to receive guidance and feedback. In addition, several participants voiced their anxiety about what was expected of the specialist paramedic, and whether they were fulfilling those requirements.

I don’t know what’s expected of me because no one has ever really made that clear. Until this is addressed I won’t know if I’m doing my job right or wrong really.

Knowledge transfer from an advanced practitioner was a frequently recurring theme whereby a willing mentor providing a mutual, supportive and respectful process was viewed as an essential component. Coaching through the development of a supportive professional relationship was also considered important in developing the personal connection in order to fully appreciate each other’s role.

Although the initial preference was for a less formal mentoring relationship, a degree of structure was also considered important in order to facilitate a direction of development and reinforce the need for the process in maintaining clinical effectiveness. All participants agreed that time and investment were required to ensure that mentorship occurred at least once a month.

## Discussion

Mentorship was viewed as an essential component in developing the specialist paramedic role, and ensuring the maintenance of clinical competence. The current model of clinical supervision was not seen as sufficient for specialist paramedics due to their different development needs and clinical practice.

It has been reported that mentorship is associated with increased levels of empowerment, role satisfaction and coaching of career goals, and may address the frustrations expressed regarding a lack of support and development (Hodgson & Scanlan, 2013). In addition, when mentorship is utilised as an assessment and feedback tool, there is a strong association with effective clinical development in both the nursing and paramedic professions (Sibson & Mursell, 2010).

The participants in this study expressed views consistent with the theory of mentorship and existing models, aligned to similar models from the nursing profession whereby an appropriate blend of formal and informal mentorship has emerged. Formal mentorship was viewed as important in order to receive organisational support and to facilitate the structure and direction of mentorship to make it an effective process. However, the informal components were also considered necessary so that only the most driven mentors with the required skills and behaviours could undertake the role in a way that developed a trusted and genuine relationship between both parties.

Similarly, the desire for the transfer of knowledge from an advanced practitioner experienced in their field meant that a degree of sponsorship mentoring was required where a more formalised approach from a senior clinician must be balanced against the developmental mentorship model of a mutually beneficial relationship of trust and value. This reflects the emergence of the blended model in literature requiring the structure and direction needed to formalise the process and the interpersonal relationships and skills required to embed a culture of caring and valuing of the clinician’s contribution towards organisational goals (Spouse, 2001).

The study participants argued that to enable the assessment and transfer of knowledge a mentorship programme should be delivered on a one-to-one basis by a more qualified and experienced practitioner. While group sessions were considered useful, it was the individual mentorship sessions in real time clinical practice that were viewed to have the most potential to maximise skills.

Furthermore, this model would also allow for clarity in role, increased motivation and role satisfaction in addition to delivering the benefits of increased clinical effectiveness and performance. The psychosocial benefit of mentorship is also considered essential in the management of stress, and viewed as important by the participants.

Since relatively few advanced paramedic roles currently exist in the UK, clinical mentorship from this staff group may be difficult to achieve. However, recent developments with paramedics rotating through primary care may provide a new opportunity for supervision and mentorship by general practitioners (Health Education England, 2018). This may lead to a new model of mentorship that sees the general practitioner and the emerging advanced paramedic providing clinical mentorship from both primary care and pre-hospital care respectively. The result may be a highly competent clinician with skills that are transferrable across different healthcare settings to the benefit of the wider health system.

### Limitations

This study has several limitations. The sample size selected was based on the time limits of the student and not the requirements to reach data saturation. In addition, the results of this study may not be generalisable to specialist paramedics across the UK working in other services. Interviews and data coding were undertaken by the student alone and, even with support provided by the senior paramedic and the university supervisor, this may have led to bias during data analysis.

## Conclusion

Ambulance services considering the implementation of specialist and advanced practice must plan for the delivery of a mentorship and clinical supervision model in order to successfully implement such roles. Due to the expanding scopes of practice of paramedics delivering extended roles, skill and knowledge decay will pose a challenge and must be addressed if outcomes such as safe and appropriate non-conveyance to ED measures are to be achieved.

However, without an appropriate mentorship structure, there is a very real danger of education being delivered to qualify clinicians as specialist paramedics, but without the robust continuous experiential learning to consolidate academic education into enhanced critical thinking practitioners in clinical practice.

This can be mitigated by implementing a model of mentorship where the advanced practitioner is able to provide individual one-to-one mentorship support. Organisations that support this would therefore need to implement a formal structure to such a model, but must be cognisant of the need to allow less formal interpersonal relationships to develop to facilitate the softer components that lead to improved motivation, a valued culture and psychological support.

## Author contributions

AH was the NHS project supervisor supporting the MSc student as host employer, and is the primary author and guarantor of manuscript. SS was an MSc dissertation student who undertook a research project on behalf of the Yorkshire Ambulance Service. JPW was the university student dissertation supervisor, reviewer and contributor to the manuscript.

## Conflict of interest

None declared.

## Ethics

Ethical approval was gained from the student’s university approval processes to undertake this study, with permission granted by the Yorkshire Ambulance Service to conduct the research interviews and report the findings.

## Funding

None.
